# Regulation of Lignin Biosynthesis and Its Role in Growth-Defense Tradeoffs

**DOI:** 10.3389/fpls.2018.01427

**Published:** 2018-09-28

**Authors:** Meng Xie, Jin Zhang, Timothy J. Tschaplinski, Gerald A. Tuskan, Jin-Gui Chen, Wellington Muchero

**Affiliations:** ^1^Biosciences Division, Oak Ridge National Laboratory, Oak Ridge, TN, United States; ^2^Center for Bioenergy Innovation, Oak Ridge National Laboratory, Oak Ridge, TN, United States; ^3^Department of Plant Sciences, University of Tennessee, Knoxville, Knoxville, TN, United States

**Keywords:** phenylpropanoid, lignin, transcription factor, growth-defense tradeoffs, secondary cell wall, transcriptional co-regulation

## Abstract

Plant growth-defense tradeoffs are fundamental for optimizing plant performance and fitness in a changing biotic/abiotic environment. This process is thought to involve readjusting resource allocation to different pathways. It has been frequently observed that among secondary cell wall components, alteration in lignin biosynthesis results in changes in both growth and defense. How this process is regulated, leading to growth or defense, remains largely elusive. In this article, we review the canonical lignin biosynthesis pathway, the recently discovered tyrosine shortcut pathway, and the biosynthesis of unconventional C-lignin. We summarize the current model of the hierarchical transcriptional regulation of lignin biosynthesis. Moreover, the interface between recently identified transcription factors and the hierarchical model are also discussed. We propose the existence of a transcriptional co-regulation mechanism coordinating energy allowance among growth, defense and lignin biosynthesis.

## Introduction

Lignin is a heterogeneous polymer of monolignols and is polymerized at the surface of the cell walls. The three essential monolignols of plant lignin are p-hydroxyphenyl (H), guaiacyl (G), and syringyl (S) units (Boerjan et al., 2003). Lignin is important for terrestrial plants by providing structural support for the upward growth of plants and enabling the long-distance water transportation, which are essential for the evolutionary adaptation of plants from the aquatic to terrestrial environment. Lignin also provides physical and chemical protection for plants against pathogen invasion.

The biosynthesis process of lignin has attracted much attention as lignin is the major contributor to the recalcitrance of biomass feedstocks (Studer et al., 2011), dramatically increasing the cost of biomass deconstruction and biofuels production. To increase the digestibility of biomass, genetic engineering of lignin has been an active research area in the past decade (Vanholme et al., 2008) and efforts have been guided toward reducing the cost of biofuel production. On the other hand, the lignin residues after biomass saccharification can be used to produce biodegradable plastic and lignin-derived value-added solvents and chemicals (Doherty et al., 2011; Ragauskas, 2016).

Given the extensive biological and industrial importance of lignin, the understanding of lignin biosynthesis in plants is beneficial for both agricultural and industrial purposes. In this review, we summarize the current understanding of the lignin biosynthesis process and its transcriptional regulation. We then offer a working hypothesis on the transcriptional coordination of energy flux among plant growth, defense, and lignin biosynthesis.

## Canonical Lignin Biosynthesis Pathway

In plants, there are two major steps to produce lignin: monolignol biosynthesis and monolignol polymerization via free radical coupling. Enzymes catalyzing monolignol biosynthesis have been well defined in the model plant *Arabidopsis*. Genetic modulation of these enzymes has been shown to dramatically alter the accumulation and/or composition of lignin.

**FIGURE 1:**
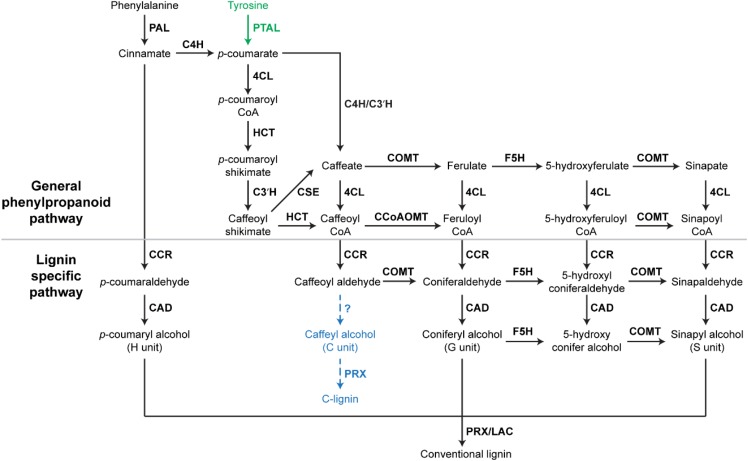
A scheme of lignin biosynthesis in plants. Black arrow indicates the canonical lignin biosynthesis in plants. Green arrow indicates the tyrosine shortcut pathway found in *Brachypodium distachyon*. Blue dashed arrow indicates the putative C-lignin biosynthesis pathway found in seed coats of vanilla orchid and most Cactoidae genera. Bold font indicates enzymes. PAL, phenylalanine ammonia-lyase; C4H, cinnamate 4-hydroxylase; 4CL, 4-coumarate: CoA ligase; HCT, quinateshikimate *p*-hydroxycinnamoyltransferase; C3′H, *p*-coumaroylshikimate 3′-hydroxylase; CCoAOMT, caffeoyl-CoA *O*-methyltransferase; CCR, cinnamoyl-CoAreductase; F5H, ferulate 5-hydroxylase; CAD, cinnamyl alcohol dehydrogenase; COMT, caffeic acid *O*-methyltransferase; CSE, caffeoyl shikimate esterase; PRX, peroxidase; LAC, laccase.

In *Arabidopsis*, monolignols are synthesized from phenylalanine via the phenylpropanoid pathway. Therefore, most key phenylpropanoid biosynthetic enzymes are also critical for lignin biosynthesis. A recent study of 221 independent transgenic *Populus* lines has demonstrated the importance of phenylpropanoid biosynthetic enzymes for lignin biosynthesis in *Populus* (Wang et al., 2018). The phenylpropanoid pathway is essential in plants, providing precursors for numerous secondary metabolites, including monolignols, flavonoids, and coumarins (Fraser and Chapple, 2011). In the phenylpropanoid pathway, three enzymes: phenylalanine ammonia-lyase (PAL), cinnamate 4-hydroxylase (C4H), and 4-coumarate: CoA ligase (4CL), catalyze the first three steps in sequence to provide precursors for all of the downstream metabolites (Fraser and Chapple, 2011). In *Arabidopsis* and *Populus*, genetic inhibition of *PAL*, *C4H*, and *4CL* genes has been shown to significantly decrease lignin content (Rohde et al., 2004; Chen et al., 2006; Vanholme et al., 2008; Wang et al., 2018). In addition to these three enzymes, other phenylpropanoid biosynthetic enzymes, such as quinate/shikimate *p*-hydroxycinnamoyltransferase (HCT), *p*-coumaroylshikimate 3′-hydroxylase (C3′H), caffeoyl shikimate esterase (CSE), caffeic acid *O*-methyltransferase (COMT), and caffeoyl-CoA *O*-methyltransferase (CCoAOMT), which work downstream of 4CL, are also indispensable for normal lignin biosynthesis (**Figure 1**). In *Arabidopsis*, the down-regulation of *HCT* and *C3′H* has been shown to induce the enrichment of H units in lignin polymers (Shadle et al., 2007; Pu et al., 2009). Similarly, the down-regulation of *HCT* gene families dramatically increased the accumulation of H units in *Populus* (Wang et al., 2018). In *Arabidopsis*, *Populus*, and *Medicago truncatula*, *CSE* mutants were shown to deposit less lignin (Vanholme et al., 2013; Ha et al., 2016; Saleme et al., 2017). COMT has been found to be critical for the synthesis of S units (Goujon et al., 2003). The *Arabidopsis* null allele of *CCoAOMT* (*ccomt1*) also exhibits reduced lignin content, as well as reduced G units (Do et al., 2007). It is notable that these early steps of monolignol biosynthesis may have variations in monocots. A systematic study of lignin biosynthesis in switchgrass has demonstrated that the conversion of *p*-coumaroyl CoA to caffeoyl CoA in switchgrass may have an alternative route, which involves the formation of quinate esters catalyzed by HCT-like enzymes (Shen et al., 2013). In addition, the direct involvement of CCoAOMT in lignin biosynthesis was questioned because the knockdown of *CCoAOMT* genes (*CCoAOMT1* and *CCoAOMT2*) did not change lignin content in switchgrass (Shen et al., 2013).

In addition to enzymes involved in the general phenylpropanoid pathway, enzymes specific for lignin biosynthesis have been identified and characterized, including cinnamoyl-CoA reductase (CCR), ferulate 5-hydroxylase (F5H), cinnamyl alcohol dehydrogenase (CAD) (Barros et al., 2015). These lignin biosynthesis-specific enzymes act downstream of phenylpropanoid biosynthetic enzymes and catalyze the biosynthesis of specific monolignols (**Figure 1**). The down-regulation of *F5H* can result in significant increase of G units in lignin (Chen et al., 2006). *Arabidopsis* mutants of CAD exhibit reduced lignin content, but increased accumulation of G and S units (Sibout et al., 2005). Besides CCR, F5H, and CAD, several lignin biosynthesis-specific steps are catalyzed by COMT, including the conversions of caffeoyl aldehyde to coniferaldehyde, 5-hydroxyl coniferaldehyde to sinapaldehyde, and 5-hydroxy conifer alcohol to sinapyl alcohol (**Figure 1**).

After biosynthesis, monolignols are polymerized to form lignin. It has been suggested that peroxidases and laccases are key enzymes catalyzing monolignol polymerization though experimental evidence is incomplete. Genetic studies of genes encoding peroxidase and laccase in *Arabidopsis* have illustrated the close relationship between these enzymes and lignin accumulation in secondary cell walls. Shigeto et al. (2015) found that double mutants of *Arabidopsis* peroxidases (*atprx2/atprx25*, *atprx2/atprx71*, and *atprx25/atprx71*) have 11–25% reduction in lignin content (Shigeto et al., 2015). Additionally, another peroxidase PRX17 has been found to be critical for lignin accumulation in a recent genetic study in *Arabidopsis* (Cosio et al., 2017). Similarly, laccases have been shown to affect lignin accumulation. A 20–40% reduction in lignin content has been observed in knockout mutants of two laccase genes (*LAC4* and *LAC17*) in *Arabidopsis* (Berthet et al., 2011). Moreover, the triple mutant of *LAC4*, *LAC11*, and *LAC17* completely lost lignin deposition in roots (Zhao et al., 2013).

## Tyrosine Shortcut Pathway

A recent study of the model grass *Brachypodium distachyon* defined a monolignol biosynthesis process with fewer steps (Barros et al., 2016). In this process, monolignols are produced from tyrosine, which is directly converted into *p*-coumarate by a grass bifunctional phenylalanine and tyrosine ammonia-lyase (PTAL) (Barros et al., 2016). Consequently, this tyrosine shortcut of monolignol biosynthesis in grasses does not contain the steps catalyzed by C4H (**Figure 1**). With fewer steps, the tyrosine shortcut pathway is energetically more efficient than the canonical lignin biosynthesis pathway. The tyrosine shortcut pathway is capable of guiding carbon and electrons into the biosynthesis of lignin via skipping the production of cinnamate, which is the essential precursor of benzenoid volatiles and salicylic acid. Therefore, the discovery of the tyrosine shortcut pathway provides an alternative approach to optimize the energy investment of lignin production. Although PTAL activity has been detected in some non-grass species, such as tobacco callus and castor bean endosperm (Gregor, 1976; Beaudoin-Eagan and Thorpe, 1985), the involvement of tyrosine shortcut pathway in lignin biosynthesis of non-grass species remains unstudied.

## C-Lignin Pathway

In addition to the H, G, and S units of lignin, a natural lignin (called C-lignin) solely containing an unusual Catechyl (C) unit was found in seed coats of vanilla orchid and most Cactoidae genera (Chen et al., 2012, 2013). In C-lignin, caffeyl alcohols are linearly connected by benzodioxane bonds via radical coupling reactions putatively catalyzed by a peroxidase (**Figure 1**; Chen et al., 2013). Although the detailed caffeyl alcohol biosynthesis and polymerization processes remain unclear, the study in dicot plants demonstrated that the biosynthesis of C-lignin and conventional lignin may be controlled differently (Tobimatsu et al., 2013). C-lignin and conventional lignin were found to be synthesized in a spatially and/or temporally separated manner in seed coats of several dicot plants (Tobimatsu et al., 2013). Without side chains, the linear lignin has less cross-linking with cellulose and hemicellulose, and is capable of enhancing the digestibility of biomass. Further understanding of the mechanism of C-lignin biosynthesis will provide an alternative bioengineering approach to generate better biomass for biofuel production. In addition, the linear C-lignin may be an ideal natural material to replace fossil-fuel based materials for the production of carbon fibers. Compared with fossil-fuel-based feedstocks, the renewable and degradable features of C-lignin make it more environmentally attractive.

## Transcriptional Regulation of Lignin Biosynthesis

**FIGURE 2:**
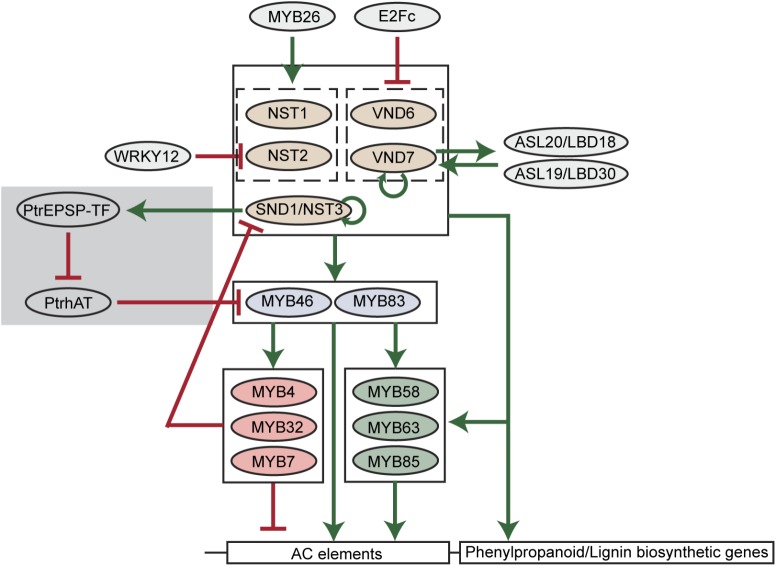
A scheme of transcriptional regulation of lignin biosynthetic genes in plants. Green arrows indicate transcriptional activation. Red blunt arrows indicate transcriptional repression. The gray shade indicates the woody plant-specific regulatory loop.

A key finding of lignin biosynthesis studies is that AC elements widely exist in the promoters of major phenylpropanoid and lignin biosynthetic genes (Rogers and Campbell, 2004). AC elements are DNA motifs rich in adenosine and cytosine. Such significant enrichment of AC elements suggests that the phenylpropanoid and lignin biosynthesis may be under the control of specific types of transcription factors. In the past two decades, key transcription factors regulating the carbon flux into phenylpropanoid and lignin biosynthesis pathways have been identified and a hierarchical transcriptional network connecting these transcription factors has been established (Nakano et al., 2015; **Figure 2**).

Among transcription factors regulating phenylpropanoid and lignin biosynthesis, MYB46 and its close homolog MYB83 are well-studied in various plant species. In *Arabidopsis*, MYB46 and MYB83 have been found to activate the expression of *PAL1*, *C4H*, *4CL1*, *C3′H1*, *HCT*, *CCoAOMT*, *CCR1*, *F5H1*, *CAD6* genes via binding to AC elements in the promoters of these genes (Zhong and Ye, 2012; Kim et al., 2014). The pine and *Eucalyptus* MYB46 homologs (PtMYB4 and EgMYB2) were also found to be functional in the regulation of lignin biosynthesis (Patzlaff et al., 2003; Goicoechea et al., 2005). Moreover, four MYB46 homologs in *Populus* (PtrMYB002, PtrMYB003, PtrMYB020, and PtrMYB021) were capable of triggering ectopic lignin deposition in *Arabidopsis* and *Populus* plants (Wilkins et al., 2009; McCarthy et al., 2010; Zhong et al., 2013). In addition to lignin, the biosynthesis of other secondary cell wall components, cellulose and xylan, can also be activated by MYB46 (Zhong and Ye, 2012). Downstream of MYB46/MYB83, multiple MYB transcription factors have been identified as specific regulators of lignin biosynthesis in *Arabidopsis*, including MYB58, MYB63, MYB85, MYB4, MYB32, and MYB7. Among them, MYB58 and MYB63 are close homologs and specifically activate lignin biosynthesis via targeting AC elements (Zhou et al., 2009). By inducing *4CL* expression, MYB85 is also capable of activating monolignol biosynthesis (Zhong et al., 2008). In contrast, MYB4, MYB32, and MYB7 were found to negatively regulate the expression of lignin biosynthetic genes (Jin et al., 2000; Preston et al., 2004; Wang and Dixon, 2012).

Upstream of MYB46/MYB83, the NAC transcription factor SECONDARY WALL-ASSOCIATED NAC DOMAIN PROTEIN 1/NAC SECONDARY WALL THICKENING PROMOTING FACTOR 3 (SND1/NST3) and its close homologs NST1, NST2, VASCULAR-RELATED NAC DOMAIN6 (VND6), and VND7 regulate lignin biosynthesis in *Arabidopsis* (Kubo et al., 2005; Mitsuda et al., 2005; Zhong et al., 2006). Similar to MYB46/MYB83, these NAC transcription factors also regulate the biosynthesis of cellulose and xylan. In *Arabidopsis*, the regulation of secondary cell wall biosynthesis by these NAC transcription factors is cell-type specific. For example, VND6 and VND7 are specifically expressed in xylem vessel cells and regulate secondary cell wall thickening. Knockdown of VND6 or VND7 using a dominant chimeric repressor specifically inhibits the formation of metaxylem and protoxylem vessels (Kubo et al., 2005). In contrast, NST transcription factors were found to preferentially regulate the secondary cell wall biosynthesis of fiber cells [SND1/NST3 and NST1, (Zhong et al., 2006; Mitsuda et al., 2007)], silique cells [SND1/NST3 and NST1, (Mitsuda and Ohme-Takagi, 2008)], and anther endothecium [NST1 and NST2 (Mitsuda et al., 2005)]. However, the cell-type specificity seems to be absent in the woody plant *Populus* and monocots, suggesting distinct regulatory mechanisms may exist. In *Populus*, both VND and NST homologs were found to be expressed in vessels and fibers, though only homologs of VND transcription factors were found to be expressed in primary xylem vessels which have no secondary growth (Ohtani et al., 2011). In monocots, such as rice and maize, homologs of VND and NST transcription factors are named SECONDARY WALL-ASSOCIATED NAC (SWN) due to their regulatory roles in the secondary cell wall biosynthesis of vessel and fiber cells (Zhong et al., 2011).

Because of the essential regulatory roles during secondary cell wall formation, the hierarchical network comprised of NAC and MYB transcription factors is thought to be the major transcriptional regulatory mechanism of lignin and secondary cell wall biosynthesis (Nakano et al., 2015). In this network, feed-forward loops are prevalent. For example, NAC transcription factors directly activate MYB46/MYB83. Meanwhile, NAC transcription factors and MYB46/MYB83 directly activate the downstream MYB58, MYB63, MYB85, and lignin biosynthetic genes (**Figure 2**). Similarly, MYB46/MYB83 directly activates MYB58, MYB63, and MYB85, and they together directly activate lignin biosynthetic genes (**Figure 2**). These feed-forward loops are probably to ensure the robust regulation of lignin biosynthesis. Besides feed-forward loops, the NAC-MYB network also contains feed-back regulations to fine-tune lignin biosynthesis (**Figure 2**). For example, SND1 and VND7 were found to directly target and activate the expression of themselves in *Arabidopsis*, representing positive feed-back regulation (Wang et al., 2011; Endo et al., 2015). A negative feed-back regulatory loop, in which SND1 is targeted and down-regulated by downstream MYB transcription factors including MYB4, MYB7, and MYB32, was also defined in *Arabidopsis* (Wang et al., 2011).

Recent discoveries of new transcription factors in *Arabidopsis*, and other plant species, suggest that the regulatory network of lignin biosynthesis in plants extends beyond the established NAC-MYB network (Rao and Dixon, 2018). Among these new transcription factors, several regulate members of the NAC-MYB network. A transcriptional repressor E2Fc has been reported to directly target *VND6* and *VND7* in a large-scale yeast one hybrid screening (Taylor-Teeples et al., 2015). The knockdown of E2Fc using RNAi induced ectopic lignin deposition in *Arabidopsis* roots (Taylor-Teeples et al., 2015), further demonstrating that E2Fc is a negative regulator of lignin biosynthesis. Moreover, *Arabidopsis* WRKY12 was found to repress lignin biosynthesis via direct repression of the *NST2* gene (Wang et al., 2010). Knockdown of WRKY12 in *Medicago* was found to enhance lignin deposition in cell walls (Wang et al., 2010). The positive regulators of SND1 and its close homologs have also been identified in *Arabidopsis*. *ASYMMETRIC LEAVES2-LIKE20*/*LATERAL ORGAN BOUNDARIES DOMAIN18* (*ASL20/LBD18*) and *ASL19*/*LBD30* genes are activated by VND6 and VND7 (Soyano et al., 2008). Overexpression of *ASL20*/*LBD18* and *ASL19*/*LBD30*, in turn, enhances the expression of *VND7*, suggesting a positive feedback loop amplifying *VND7* expression (Soyano et al., 2008). In addition, overexpression of MYB26 was found to increase lignin deposition and the expression of *NST1* and *NST2* (Yang et al., 2007). The discovery of these positive and negative regulators demonstrates that the NAC-MYB network is regulated to spatially and/or temporally switch on or off lignin biosynthesis.

Using genome-wide association studies (GWAS), a novel transcription factor was identified that regulates the expression of *PtrMYB021* (homolog of *MYB46*) in *Populus* (Xie et al., 2018). This transcription factor (PtrEPSP-TF) is a *Populus* homolog of 5-enolpyruvylshikimate 3-phosphate (EPSP) synthase, an enzyme catalyzing the shikimate pathway. Aside from the well-established enzyme function of EPSP synthase, PtrEPSP-TF can act as a transcriptional repressor. Genetic and molecular studies further revealed that PtrEPSP-TF activates *PtrMYB021* expression and lignin biosynthesis by inhibiting the expression of a transcriptional repressor of *PtrMYB021*, which is named PtrhAT (Xie et al., 2018). Different from the ancestral EPSP synthase, the PtrEPSP-TF protein contains an additional helix-turn-helix (HTH) motif at its N-terminus, which is indispensable for the nuclear accumulation and transcriptional function of PtrEPSP-TF (Xie et al., 2018). However, the HTH motif is almost entirely missing in EPSP synthases of non-vascular plants, algae, and monocots (Xie et al., 2018). As opposed to herbaceous plants (e.g., *Arabidopsis*), woody perennial plants (e.g., *Populus*) have extensive secondary cell wall thickening over multiple growing seasons. The discovery of the additional regulatory loop of *MYB46* in *Populus* supports the existence of woody plant-specific regulatory mechanisms in lignin biosynthesis. Moreover, the discovery of an activator (PtrEPSP-TF) and repressor (PtrhAT) of *MYB46* provides alternative approaches to fine tune lignin biosynthesis.

## Lignin Biosynthesis Has Complex Crosstalk With Growth and Defense

Crosstalk among biological processes is widespread. Genetic studies in *Arabidopsis* have demonstrated crosstalk among lignin biosynthesis, growth, and defense. Meanwhile, the complexity of the crosstalk is demonstrated by these studies. To date, the relationship of lignin content with growth and defense remains unpredictable due to the limited understanding of the underlying mechanisms.

Lignin is thought to be indispensable for plant growth. The disruption of lignin biosynthesis by knocking down lignin biosynthetic genes, such as *C4H* and *CCR1*, was found to co-occur with the suppression of growth rate (Vanholme et al., 2012). However, recent studies suggest that lignin-growth crosstalk is more complex. A study of one *C3′H* mutant (*ref8-1*) illustrated that the growth suppression of *ref8-1* might be due to the flavonoid hyperaccumulation, rather than the impaired lignin biosynthesis (Besseau et al., 2007; Bonawitz et al., 2014). On the other hand, increased lignin accumulation is also harmful for plant growth. The ectopic lignin deposition induced by the overexpression of NAC and MYB transcription factors, such as SND1, MYB46/83, and MYB58/63, was observed together with inhibited growth in *Arabidopsis* (Zhong et al., 2006, 2007; Zhou et al., 2009).

The crosstalk between lignin biosynthesis and defense is even more complicated. Lignin is a well-known defense polymer, which has antimicrobial activity, forms a physical barrier to block pathogen invasion, and prevents the ingress or diffusion of toxins from pathogens (Sattler and Funnell-Harris, 2013). It has been widely observed that lignin deposition and lignin biosynthetic genes are induced during the attack of various pathogens. However, the genetic suppression of multiple lignin biosynthesis-related genes was shown to enhance pathogen resistance. For example, *Arabidopsis MYB46* mutants (with impaired lignin biosynthesis) displayed enhanced disease resistance, which was thought to be caused by cell wall integrity damage-triggered high immunity level (Ramírez et al., 2011). The repression of *HCT* in *Arabidopsis* and *Medicago* was found to trigger the accumulation of defense hormone salicylic acid (SA) and the expression of *PATHOGENESIS-RELATED* (*PR*) genes (Gallego-Giraldo et al., 2011a,b).

Studies on the *Arabidopsis HCT* mutant further illustrated the crosstalk among lignin biosynthesis, growth, and defense. *HCT* mutant has reduced lignin content, stunt growth, and enhanced defense (Gallego-Giraldo et al., 2011a). The reduced lignin content was found to trigger the accumulation of SA and the expression of *PR* genes, which subsequently enhance the pathogen resistance (Gallego-Giraldo et al., 2011a). Impaired lignin biosynthesis is generally thought to cause growth deficiency. However, in *HCT* mutant, the stunt growth was found to be unrelated with the reduced lignin content, but caused by the increased SA level (Gallego-Giraldo et al., 2011a).

## The Transcriptional Co-Regulation of Lignin Biosynthesis, Growth, and Defense

Transcriptional co-regulation has been identified as a mechanism balancing growth and defense. In a recent study, Campos et al. (2016) found that transcriptional networks downstream of jasmonic acid (JA) and phytochrome B signaling form a hub to coordinate growth and defense. The down-regulation of both JA and phytochrome signaling (*jazQ phyB* double mutant) has been shown to uncouple growth and defense, resulting in plants displaying more growth and more defense (Campos et al., 2016).

Besides the co-regulation of growth and defense, evidence has accumulated that indicates the possible existence of transcriptional co-regulatory mechanisms coordinating growth and lignin biosynthesis, as well as defense and lignin biosynthesis. For example, the semi-dominant mutant (*ref4-3*) of one subunit of the transcriptional co-regulatory complex Mediator (MED5b) was found to exhibit the dwarfism phenotype, as well as repressed phenylpropanoid and lignin production (Bonawitz et al., 2012). Disruption of *MED5b* is capable of rescuing both the growth and lignin biosynthesis deficiencies of the C3′H mutant (Bonawitz et al., 2014), further demonstrating the involvement of transcriptional mechanisms in the co-regulation of growth and lignin biosynthesis. On the other hand, several transcription factors have been found to affect lignin biosynthesis, as well as defense responses. Overexpression of one *Medicago* WRKY transcription factor gene *WRKY W109669* in tobacco was shown to enhance the accumulation of lignin and the transcription of *PATHOGENESIS-RELATED 2* (*PR2*) gene (Naoumkina et al., 2008), which is a defense gene induced in response to virus infection (Ward et al., 1991). A recent genetic study of a *Gossypium barbadense* ethylene response-related factor gene (*GbERF1-like*) also demonstrated the transcriptional co-regulation of defense and lignin biosynthesis. Overexpression of *GbERF1-like* in *Arabidopsis* significantly enhanced the transcription of phenylpropanoid/lignin biosynthetic genes (e.g., *PAL3*, *C4H*, *C3′H*, *HCT*, *CCoAOMT1*, *CCR1*), as well as pathogenesis-related genes including *PR3*, *PR4*, and *PLANT DEFENSIN1.2* (*PDF1.2*) (Guo et al., 2016). Moreover, the *Arabidopsis myb46* mutants were found to be resistant to the necrotrophic pathogen *Botrytis cinerea* and to increase *PR3* and *PDF1.2* expression after *Botrytis cinerea* infection (Ramírez et al., 2011).

More importantly, genetic studies in *Arabidopsis* have demonstrated the possibility of the transcriptional co-regulation of growth, defense, and lignin biosynthesis. In mutants of *C4H* and *CCR1*, which display significantly reduced lignin accumulation, many defense-responsive genes and many growth-related genes (i.e., auxin response genes) are down-regulated (Vanholme et al., 2012). A MADS-box transcription factor AGAMOUS-LIKE15 (AGL15) was found to regulate the expression of miRNA156 (Serivichyaswat et al., 2015), which negatively regulates inflorescence development and positively regulates tolerance to recurring environmental stress (Wang et al., 2009; Stief et al., 2014). A recent study further identified the regulatory role of AGL15 in lignin biosynthesis. In a study by Cosio et al. (2017), AGL15 was found to directly repress *PRX17* expression to regulate lignin formation.

Collectively, genetic studies have illustrated the existence of the transcriptional co-regulation mechanism coordinating growth, defense, and lignin biosynthesis. It is generally recognized that changes in lignin biosynthesis affect the structure and integration of cell walls, which in turn affects growth and defense. However, little is known about the genetic factors co-regulating growth, defense, and lignin biosynthesis or factors bridging the transcriptional co-regulations of growth-lignin biosynthesis and defense-lignin biosynthesis. This is a much-needed research area for future studies.

## A Proposed Strategy to Mitigate Growth-Defense Tradeoffs by Manipulating Lignin Biosynthesis

Growing in a dynamic environment, plants have evolved sophisticated strategies to determine the energy allowance between growth (to compete for light) and defense (to fight against pathogens). The competition for energy suggests that growth and defense of plants may have a negative relationship, which means the activation of defense processes negatively affect the growth and reproduction of plants. Such tradeoffs between growth and defense have been observed in many studies (Agrawal et al., 2012; Züst et al., 2015). Although the tradeoff between growth and defense is fundamental for plant survival in a changing environment, it is detrimental for plant yield.

Lignin biosynthesis is traditionally thought as one indivisible part of plant growth and defense, to provide structural support, transport water, and act as physical barrier. However, discoveries of transcriptional mechanisms underlying the crosstalk of lignin biosynthesis, growth, and defense suggest the possibility to unlock lignin biosynthesis from growth and defense. On the other hand, lignin accounts for approximately 30% of the organic carbon in the biosphere (Cesarino et al., 2012). This large amount of energy invested in lignin and its precursors has the potential to compensate the costly expenditure of defense, which consequently would mitigate the tradeoff between growth and defense. Collectively, the genetic modification of the co-regulatory mechanism represents a potential strategy to overcome growth-defense tradeoffs.

## Prospective

To date, the lignin biosynthesis process and its regulatory mechanism in the model plant *Arabidopsis* are well established. Although the lignin biosynthesis process has been well-studied and monolignol biosynthetic enzymes have been systematically analyzed in woody plants, such as *Populus*, the regulation of lignin biosynthesis remains poorly understood. The regulatory mechanism in a woody perennial plant is much more complex than that in *Arabidopsis*, due to the complex genome and long life-cycle. With the application of genome-wide approaches, such as GWAS, expression quantitative trait loci (eQTL), and expression quantitative trait nucleotide (eQTN) mapping, in plant studies, it is plausible to identify causal genes affecting complex traits including lignin biosynthesis. Together with efficient post-GWAS characterization and validation, species-specific mechanisms of lignin biosynthesis can be defined.

The concept of a transcriptional hub to coordinate carbon and energy flux into growth, defense, and lignin biosynthesis is just emerging based on studies in *Arabidopsis*. A comprehensive understanding of this transcriptional hub will have significant impacts on the field of bioengineering. Current studies of NAC-MYB network are restricted in secondary cell wall biosynthesis, although RNA-seq analyses have illustrated that NAC and MYB transcription factors can induce broader gene expression changes. By identifying genome-wide targets of secondary cell wall-related NAC and MYB transcription factors using ChIP-seq, molecular mechanisms linking lignin biosynthesis, growth, and defense are expected to be discovered. In the woody plant *Populus*, although data is relatively limited, our recent study demonstrated the transcriptional co-regulation of lignin biosynthesis and defense. A defense-responsive WRKY transcription factor was found to regulate the expression of a *Populus HCT* gene (*PtHCT2*) (Zhang et al., 2018). With the identification and characterization of transcription factors with multiple functions in the regulation of growth, defense, and lignin biosynthesis, a transcriptional network needs to be established to unveil how growth, defense, and lignin biosynthesis are co-regulated. With this knowledge, the resource flux in plants can be fine-tuned depending on human demand, which will greatly reduce the cost of agricultural and forestry biofuels production.

## Author Contributions

MX drafted the manuscript. JZ, TT, GT, J-GC, and WM revised the manuscript.

## Conflict of Interest Statement

The authors declare that the research was conducted in the absence of any commercial or financial relationships that could be construed as a potential conflict of interest.
